# Extraction Efficiency of Different Solvents and LC-UV Determination of Biogenic Amines in Tea Leaves and Infusions

**DOI:** 10.1155/2016/8715287

**Published:** 2016-07-31

**Authors:** U. Gianfranco Spizzirri, Nevio Picci, Donatella Restuccia

**Affiliations:** Department of Pharmacy, Health and Nutritional Sciences, University of Calabria, Edificio Polifunzionale, 87036 Arcavacata di Rende, Italy

## Abstract

Biogenic amines (BAs), that is, spermine, spermidine, putrescine, histamine, tyramine, *β*-phenylethylamine, cadaverine, and serotonin, have been determined in several samples of tea leaves, tea infusions, and tea drinks by LC-UV method after derivatization with dansyl chloride. Different extraction solvents have been tested and TCA 5% showed better analytical performances in terms of linearity, recovery percentages, LOD, LOQ, and repeatability than HCl 0.1 M and HClO_4_ 0.1 M and was finally exploited for the quantitative determination of BAs in all samples. In tea leaves total BAs concentration ranged from 2.23 *μ*g g^−1^ to 11.24 *μ*g g^−1^ and PUT (1.05–2.25 *μ*g g^−1^) and SPD (1.01–1.95 *μ*g g^−1^) were always present, while SER (nd–1.56 *μ*g g^−1^), HIS (nd–2.44 *μ*g g^−1^), and SPM (nd–1.64 *μ*g g^−1^) were detected more rarely. CAD and PHE were determined in few samples at much lower concentrations while none of the samples contained TYR. Tea infusions showed the same trend with total BAs concentrations never exceeding 80.7 *μ*g L^−1^. Black teas showed higher amounts of BAs than green teas and organic and decaffeinated samples always contained much lower BAs levels than their conventional counterparts.

## 1. Introduction

Tea (*Camellia sinensis*) consumption is rooted in medicinal use in China five thousand years ago. Since then, it has become the world's most popular drink (after water), whose industry employs more than 13 million people around the world. The tea crop has rather specific agroclimatic requirements that are only available in tropical and subtropical climates, while some varieties can tolerate marine climates of British mainland and Washington area of the Unites States. It follows that tea is primarily produced in Asia and Africa, with China, India, Kenya, Sri Lanka, and Turkey accounting for 76 percent of global production [1]. Unlike coffee and cocoa, the majority of tea production is consumed locally, in domestic markets. Nevertheless, about 40% of global production was destined for export in 2011, worth US $ 6.6 billion [1].

Today tea is available for consumption in many varieties, based on the oxidization and fermentation technique applied. Generally, tea can be broadly classified according to its production method as either unfermented (green tea), semifermented (oolong tea), fully fermented (black tea), or postfermented (Pu'er tea) [2]. However, in recent years, to support the expansion of the demand, diversification into other segments of the market has been widely encouraged, with greater attention to the sustainability. To this regard, major standards active in the tea sector include Fairtrade International, Organic, Rainforest Alliance, the Ethical Tea Partnership, and UTZ Certified. Together, these initiatives certified or verified 12 percent of global production by 2011/2012 with Kenya, India, and Malawi as the biggest producer and about one-third of production is actually sold compliant with voluntary sustainability standards on the international market [1].

Many beneficial health effects have been related to tea consumption. Bioactive compounds of this beverage, in particular polyphenolic constituents, deeply influence its antioxidative, anti-inflammatory, antimicrobial, anticarcinogenic, antihypertensive, and neuroprotective properties [3, 4]. However, it is important to state that tea contains less studied bioactive compounds, such as biogenic amines (BAs) which are nonvolatile amines formed by decarboxylation of amino acids. Natural polyamines are present at low levels in microorganisms, plants, animals, and humans where they are implicated in important physiological functions [5]. In foods, the decarboxylation process can be related to the activity of decarboxylase enzymes which are widely distributed in spoilage and other microorganisms, for example, in naturally occurring and/or artificially added lactic acid bacteria involved in food fermentation [6, 7]. Moreover, it has been reported that the oxidative decarboxylation of corresponding amino acid can be also obtained during thermal processing of foods, suggesting a new “thermogenic” formation pathway of biogenic amines [8–10]. As the consumption of food containing large amounts of these amines can have toxicological consequences, it is generally assumed that they should not be allowed to accumulate. In fact, if BAs levels in foods or beverages reach a critical threshold they may induce headaches, respiratory distress, heart palpitations, hypo- or hypertension, and several allergenic disorders [6, 11]. It follows that the determination of biogenic amines in fresh and processed foods is of great interest not only due to their toxicity, but also because they can be a useful index of spoilage or ripening.

Analytical determination of biogenic amines in foods is not simple because of the complexity of the real matrices to be analyzed and the low concentration levels at which the compounds are generally present. Several methods have been developed for determination of BAs in food which are mainly based on chromatographic techniques coupled with UV, fluorimetric, mass spectrometry, and evaporative light scattering detection [12–14]. Among them, LC coupled with UV detection is at the moment the reference method in Europe for histamine determination in fresh and treated fishery as dansyl derivative [15]. However, while a great number of studies are present in literature dealing with the optimization of the derivatization reaction and/or with the improving of the chromatographic performances of the methods [12], less attention is generally devoted to the pretreatment procedure of food samples which is very important in BAs analysis as well.

Preclean-up protocol comprises extraction of BAs from the sample with a suitable extracting solvent. The complexity of the varied food matrices is the most critical aspect to take into consideration during the solvent selection in order to obtain adequate recoveries for all amines. Moreover, the different handling of the food matrix makes an effective comparison of the literature data quite difficult.

Reported extraction procedures consist of the use of acids (trichloroacetic acid, hydrochloric, perchloric, thiodipropionic, or methanesulfonic acids) and solvents (petroleum ether, chloroform, or methanol) depending on the matrix [12, 13]. Anyway, extraction optimization studies have been published only for the amine contents in cheese, underlining that the extraction efficiency varies widely among amines and is affected by the levels of amines in the matrix, the type, concentration, and temperature of the solvent used [16, 17].

Considering that very few studies are present in literature dealing with BAs determination in tea or its aqueous infusions [18–23] and none of them considers recovery values of BAs obtained with different solvents, the object of the present study is the optimization of the extraction procedure of BAs from tea leaves using HCl 0.1 M, HClO_4_ 0.1 M, and TCA 5%. Moreover, after sample handling optimization, the quantitative determination of BAs in tea leaves, infusions, and tea drinks by LC-UV with dansyl derivatization has been accomplished as well.

## 2. Materials and Methods

### 2.1. Samples

Twenty-one samples of teas have been selected in grocery stores in Cosenza, Italy. Specifications of teas (country and region of origin, trade names, types of teas, and further characterization) of the considered samples, as declared by producers and/or reported on the labels, are summarized in Table 1.

Commercial tea beverages were all lemon-scented drinks obtained from conventional black teas coming from Sri Lanka. Caffeine content was not reported on the label.

To quantify BAs extractable with water, tea infusions were prepared referring to International Organization for Standardization: ISO 3103 entitled “Tea-Preparation of liquor for use in sensory test” (ISO 3103). To tea leaves (2.00 g) 70 mL of double boiling distilled water was added. After 20 min the infusion was filtered, and the cooled filtrate filled up in a volumetric flask with water to 100 mL. (Note: only single extraction of tea leaves was performed since in second extracts with hot water no polyamines could be detected by LC. This is also in agreement with common practice for preparing tea as beverage.)

### 2.2. Chemicals

BAs spermine (SPM, tetrahydrochloride), spermidine (SPD, trihydrochloride), putrescine (PUT, dihydrochloride), histamine (HIM, dihydrochloride), tyramine (TYR, hydrochloride), *β*-phenylethylamine (PHE, hydrochloride), cadaverine (CAD, hydrochloride), and serotonin (SER, hydrochloride) as well as dansyl chloride, ammonia (30%), trichloroacetic acid, and LC solvents (acetonitrile and methanol LC grade) were purchased from Sigma-Aldrich (Milford, MA, USA). Ultrapure water was obtained from Milli-Q System (Millipore Corp., Milford, MA, USA). Filters (0.45 and 0.20 *μ*m) were purchased from Sigma-Aldrich. SPE C_18_ cartridges (0.5 g) were obtained from Supelco Inc. (Bellefonte, PA, USA).

### 2.3. Amine Standard Solutions and Calibration

Calibration was accomplished for LC-UV confirmation experiments. An individual standard solution of about 1.0 mg mL^−1^ of each amine was prepared in purified water and stored in darkness at 4 ± 1°C. Different aliquots of each solution were then pooled to prepare twelve BAs standard mix solutions reaching a final volume of 25 mL employing HCl 0.1 M, HClO_4_ 0.1 M, or TCA 5% (w/w). The final amine concentrations injected were 0.1, 0.5, 0.8, 2.0, 4.0, 5.0, 10.0, 16.0, 25.0, 50.0, 75.0, and 100.0 *μ*g mL^−1^. The identification of the amines was performed by comparing the retention times of peaks in the samples with those of standard solutions and by addition of the suspected amine to the samples. A calibration plot, reporting the peak area against standard concentration, was constructed for twelve concentration levels and six independent replicates for each concentration level were performed. To evaluate the matrix effect, besides external calibration plots (peak area versus concentration of standard solutions) and standard addition method plots (peak area versus concentration of standard solutions added to the sample) were built and compared. The slopes of the two plots were not significantly different, indicating no significant matrix effect. Quantitative determination was then accomplished by direct interpolation in the external calibration plot of each amine.

### 2.4. BAs Extraction and Purification

The extraction of BAs from tea power samples was performed by adding 10 mL of HCl 0.1 M or 10 mL of HClO_4_ 0.1 M or 10 mL of TCA 5% (w/w) to about 2.0 g of sample, in a 50.0 mL test tube. The mixture was homogenized (vortex at 40 Hz for 40 min), centrifuged (10,000 ×g for 20 min), filtered (syringe filter 0.20 *μ*m), collected in a plastic vial, and purified by SPE on C_18_ sorbent (conditioning: 2 mL of H_2_O and 2 mL (two times) of CH_3_OH; loading: 5.0 mL of the basified sample; washing: 2.0 mL of NH_4_OH at pH 11.0; eluting: 2.0 mL (two times) of CH_3_OH). The eluting solution was dried with nitrogen gas and the residue was redissolved in a plastic test tube with 1.3 mL of extraction solvent.

To an aliquot (40 mL) of the infusion teas,* n*-BuOH (5 mL) was added and the mixture was evaporated to dryness using a vacuum rotary evaporator. The remaining residue, dissolved in the extraction solvent (4.0 mL) and stirred for 24 h, was centrifuged and the supernatant (2 mL) was basified with NaOH 1 N and subsequently analyzed.

Recovery experiments were performed spiking, before the extraction procedure, sample 5 with an aliquot of BA standard mixture. In particular, 2 g of power tea was spiked with 1.0 mL of BA standard solution 2.0 mg L^−1^, while 40 mL of tea infusion was spiked with 5.0 mL of BA standard solution 2.0 mg L^−1^. Method validation was obtained in terms of linearity, recovery percentages, LODs, LOQs, and intra- and interday repeatability in order to ensure analytical suitability.

For dansylation reaction, at 1.0 mL of standard solution (or acid sample extract spiked with BAs or acid sample extract) 200 *μ*L of NaOH 2.0 M, 300 *μ*L of saturated NaHCO_3_ solution, and 2.0 mL of dansyl chloride solution (10.0 mg mL^−1^ in acetone prepared just before use) were added. After the reaction time (30 min at 60°C), the excess of dansyl chloride was removed by adding 100 *μ*L of NH_4_OH 25% (v/v). After filtration with 0.45 *μ*m syringe filters, a volume aliquot of 20 *μ*L was injected for LC-UV analysis.

### 2.5. Chromatographic Conditions

Liquid chromatography was performed with a Jasco PU-2080 instrument equipped with a Rheodyne 7725 injector with a 20 mL sample loop and a gradient pump (PU-2089 plus, Jasco Inc., Easton, MD, USA). The system was interfaced with UV detector operating at *λ* = 254 nm (UV-2075, Jasco Inc., Easton, MD, USA). Data were collected and analyzed with an integrator Jasco-Borwin1. A reverse-phase C_18_ column (250 mm × 4.6 ID, 5 mm) (Supelco Inc., Bellefonte, PA, USA) equipped with C_18_ guard-pak (10 mm × 4.6 ID, 5 mm) was used (Supelco Inc., Bellefonte, PA, USA) for separation of BAs. Two solvent reservoirs containing (A) purified water and (B) acetonitrile were used to separate all the amines with a gradient elution which began with 3 min of isocratic program A-B 50 : 50 (v/v) reaching after 20 min A-B 10 : 90 (v/v). Then 3 min of isocratic elution was carried out and 4 min further was necessary to restore again the starting conditions (A-B 50 : 50, v/v). Flow was kept constant at 1.2 mL min^−1^.

### 2.6. Statistical Analyses

All analyses were performed in triplicate and data were expressed as mean ± relative standard deviations (RSD). Studies of the correlation coefficient and linear regression, assessment of repeatability, calculation of average, standard deviation, and RSD were performed using Microsoft Excel 2010 software. Significance was performed using a one-way analysis of variance (ANOVA) test, employing Duncan's multiple range test at significance level *p* < 0.05.

## 3. Results and Discussion

### 3.1. Extraction Optimization and Method Performances

According to the literature, there is still no consensus on which extractor is the most appropriate for the extraction of BA from food matrix prior to LC analysis. Usually the matrix plays a crucial role as far as the levels of BAs, the type, concentration, and temperature of the solvent varied significantly the extraction efficiency. Due to the high level of BAs produced during fermentation processes, selected foodstuffs such as dairy products and meat derivatives were deeply investigated and detailed extraction information is available [16, 24, 25]. Different solvents were proposed, including water, ethanol, and methanol. The employing of acids such as HCl, HClO_4_, TCA, or sulfosalicylic [26] or employing buffers at alkaline pH [27] was also possible.

In this work, different acidic medium was tested as extraction solvents in the determination of the BAs present in the tea leaves and infusions. In particular, HCl 0.1 N, HClO_4_ 0.1 N, and TCA 5% (w/w) were proposed and the recorded results are summarized on Table 2. Linearity was observed in the whole concentration range showing for each compound good regression coefficients values. LODs for standard solutions were calculated from the amount of amines required to give a signal-to-noise ratio of 3, while LOQs were obtained considering a signal-to-noise ratio of 10. Good LOQs values were obtained by LC-UV as compared with other studies [9, 28]. LOD and LOQ values referred to the samples (leaves and infusions) expressed in *μ*g g^−1^ or *μ*g L^−1^ were also determined and derived from LOD and LOQ values relative to standard solutions, considering all handling steps during sample preparation. As can be seen, the LC-UV method is sufficiently sensitive for quantitative determination of BAs in all samples, for both the tea leaves and tea infusions.

In order to facilitate the decision on which are the best conditions for the extraction of the amines from the tea matrices, the criteria established by the Codex Alimentarius (1993) were used: percent recoveries from 80% to 115% and coefficient of variation (CV) lower than 15% [28]. As reported on Tables 3 and 4, for all BAs both parameters are in the range indicated in literature, with some slight differences. With the exception of PUT (the same values were recorded), the analysis of CVs of tea leaves displays the better CV values of TCA compared to the others extraction solvents (Table 3). In particular, for CAD and SER, CV values were observed three times lower, while for SPD and HIS, CV values were observed approximatively two times. TYR was not detected in the food matrix with all tested extraction solvents. The same trend was observed for the infusion but, in this case, two BAs (PHE and TYR) were absent in the food matrix. The choice of TCA as elected solvent was confirmed by comparing the data of recovery experiments reported in Table 4.

Because recovery depends on the concentration level of the analyte in the matrix (Miller & Miller 2000), a previous qualitative and quantitative evaluation of BAs' content was performed in the considered samples and the native amounts were so evaluated. BA standard solutions at concentrations comparable with those quantified were added and the samples were subjected to the whole treatment of SPE, dansylation, and LC-UV analysis. The recovery was evaluated for each BA by comparing the amount found after spiking (with respect to that initially estimated) and the amount added. Recovery experiments provided satisfactory percent of recoveries (>88%) for all the BAs using the three tested extraction solvents, both in the leaves and in the infusion, but excellent values were recorded employing the TCA (>97.5%). These findings suggested that the extraction with TCA at the concentration of 5% (w/w) allowed adequate extraction of most of the amines from leaves and infusion of tea.

The repeatability (intra- and interday analysis) was verified by evaluating the relative standard deviation values for peak areas measured for six repeated injections of the same sample extract; data reported on Tables 3 and 4 indicate an acceptable precision for all BAs analyzed.

**Figure 1:**
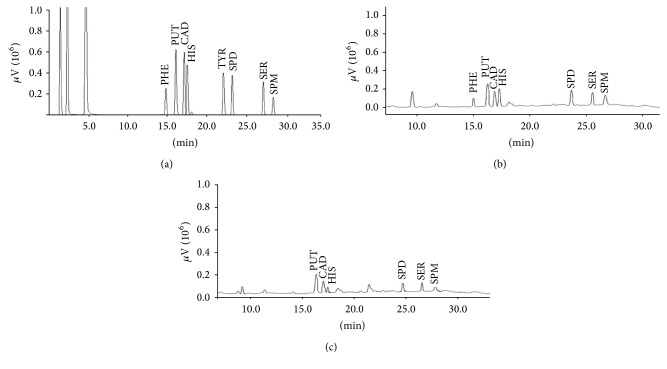
Chromatogram of BAs standard mix (75.0 *μ*g mL^−1^) (a) and of a tea leaves extract (sample 5) and of a tea infusion extract (sample 5) (b and c), obtained by LC-UV. The resolution was carried out under gradient conditions as specified in materials and method section. PHE, *β*-phenylethylamine; PUT, putrescine; CAD, cadaverine; HIS, histamine; TYR, tyramine; SPD, spermidine; SER, serotonin; SPM, spermine.

Figure 1(a) shows the chromatogram of BAs standard solution, while Figures 1(b) and 1(c) show the chromatograms of tea leaf sample and tea infusion, respectively, obtained employing TCA 5% (w/w) as extraction solvent.

### 3.2. Levels of BAs in Tea Leaves

In Table 5 the concentration values expressed in *μ*g g^−1^ of BAs in tea leaves are reported. Quantities of total BAs ranged from 2.23 *μ*g g^−1^ in sample 18 to 11.24 *μ*g g^−1^ in sample 5 which are in agreement with other studies [11, 18, 19, 21]. PUT (1.05–2.25 *μ*g g^−1^) and SPD (1.01–1.95 *μ*g g^−1^) were determined in all samples, while SER (nd–1.56 *μ*g g^−1^), HIS (nd–2.44 *μ*g g^−1^), and SPM (nd–1.64 *μ*g g^−1^) were present more rarely. CAD (nd–1.41 *μ*g g^−1^) and PHE (nd–2.52 *μ*g g^−1^) were found in very few samples while TYR concentration was always under the limit of detection irrespective of the analyzed sample. This trend is not surprising as polyamines, spermidine, and putrescine in particular are generally found in foods of vegetable origin, while CAD, TYR, and HIS are generally considered quality markers of animal products such as meat, fish, and meat derivatives [6].

It has been reported that tea generally contains polyamines [29–31], although leaves processing strongly influenced BAs levels and distributions [19, 21]. In fact, green and black tea production are markedly different. Fresh tea leaves usually undergo heating or steam treatment and fast drying to produce green tea. On the contrary, during black tea production the leaves of* Camellia sinensis* are subjected to a sequence of procedures such as weathering, destruction of plant tissues by various rolling, crushing and/or tearing processes followed by enzymatic maturation, and final drying. This nonmicrobial process, called fermentation, implies enzymatic and chemical oxidative reactions responsible for BAs formation and/or increase which cannot take place during green tea production as fast drying determines a total enzyme inactivation and no other oxidative reactions can then occur. In particular, Palavan-Ünsal et al. (2007) reported that SPM content decreased significantly during the manufacture of black tea [19], while PUT and SPD levels temporarily increased during withering and rolling and then decreased during fermentation and drying. Moreover, as reported for other foods [32–34] it should be underlined that the longer the production process, the higher the possibility of external microbial contamination leading to a further BAs accumulation. Data collected in Table 5 confirmed these findings, as black teas (samples 1–14) showed higher BAs quantities than green teas (samples 15–21).

In Table 5 it can be also seen that decaffeinated black teas (samples 9–13) and instant green teas (samples 18 and 19) generally showed lower amounts of BAs than regular black and green teas probably in relation to the industrial processes involved in the decaffeination and the soluble tea technology. Although no other studies are available in literature to accomplish any comparative evaluation of the data, the same findings have been already reported for decaffeinated and instant coffee [32, 35].

Finally it can be stated that, as already obtained for cocoa and coffee [34–36], organic samples (8, 14, 20, and 21) showed lower BAs amounts in comparison with their conventional counterparts (1–7 for black teas; 15–19 for green teas). This trend can be underlined for both black and green teas. This aspect can be firstly related to the different agricultural practices producing organic or conventional tea. Organic farming requires rigorous application of prescribed standards with strict credible certification and inspection regimes. To this regard, a lower concentration in organic tea of free amino acids which are the substrate for the formation of BAs has been reported. This is probably due to the lower mineral nitrogen contents in soils under organic management [37]. Moreover, field harvesting and leaf transport should be optimized to ensure that all harvested leaves are acceptable for tea manufacture. Factory systems need to ensure that high quality teas are produced all the time and all must be within legal or trade standard limits for microbial contamination. While bigger manufactures may rely on mechanical processing to create large batches of lower-quality blended teas, organic cultivation uses processing techniques to produce a bold, flavorful organic tea with unique qualities that cannot be found in the blended products. As the hygienic conditions of raw material as well as postharvest, fermentation, and transport processes are all key points in relation to BAs formation and increase, it could be hypothesized that organic farming rules coupled with the strict quality control along the whole production chain can limit the accumulation of BAs.

### 3.3. Levels of BAs in Tea Infusions

In Table 6 levels of BAs in tea infusions and drinks are reported as expressed in *μ*g L^−1^ as well as *μ*g g^−1^ of dry leaves (samples 1–21). It can be noted that the same trend observed for tea leaves in terms of BAs profiles was confirmed, although at much lower concentrations. In infusions BAs distribution varied as follows: PUT (10.2–21.0 *μ*g L^−1^), SPD (6.3–11.1 *μ*g L^−1^), HIS (nd–20.0 *μ*g L^−1^), SER (nd–9.1 *μ*g L^−1^), SPM (nd–10.3 *μ*g L^−1^), and CAD (nd–14.0 *μ*g L^−1^). PHE was found only in one sample, while TYR remained always undetected. Total BAs content never exceeded 80.71 *μ*g L^−1^ (sample 5) corresponding to 4.05 *μ*g g^−1^ of dry leaves. Obtained data are lower than those obtained by Brückner et al. (2012) [21] and in the same order of magnitude of those reported by Okamoto et al. (1997) and Nishimura et al. (2006) [18, 38] although different samples characteristics and analytical approaches limit the comparison of the data.

Data collected in Table 6 showed that, inside each class, the same distinctions evaluated for tea leaves can be generally recognised, meaning that beverages made with green teas show less amount of BAs than those obtained with black teas and, at the same time, decaffeinated, instant, and organic infusions contain less BAs than conventional teas. Tea drinks (samples 22–24 in Table 6) showed the lowest BAs concentrations among liquid samples which is not surprising, considering that the tea infusion is much diluted with water during the drink production process.

From comparison of corresponding data reported in *μ*g g^−1^ in Tables 5 and 6 a drastic reduction of BAs moving from leaves to tea infusions can be noted, but to a differ extent underlying that each amine can be differently sensitive to the water extraction during the beverage preparation. In particular best extracted amines by hot water in decreasing order were PUT (50.2% mean value), CAD (48.6% mean value), SER (45.2% mean value), HIS (35.4% mean value), SPM (31.1% mean value), and SPD (29.8% mean value). PHE was found in four samples of tea leaves but only in one infusion was detected and only 4% of the total present in leaves was extracted by water. The other three samples of tea leaves which contained less PHE amounts, in fact, produced infusions with PHE concentrations below the limit of detection owing to the scarce extraction efficiency of water for this amine. The lower BAs concentration in water infusions is not surprising and it has been already reported for coffee [32]. This aspect is related to the water possibility to extract only BAs in the free form, while amines are present in processed plants or foodstuffs also in bound and conjugated forms with other molecules, like polyphenols, phenolic acids, proteins, or nucleotides. These forms can be released by acid extraction and also in this case conjugates occur in both soluble and insoluble forms. As already reported for coffee and cocoa [39, 40], treatment of tea leaves with strong acids such as TCA, HClO_4_, or HCl surely increases the quantities of extractable BAs [41], although considering tea or coffee as beverages, only BAs soluble in hot water are of dietary importance.

## 4. Conclusions

The application of the LC-UV method described in this study permitted accurate and precise determination of up to 8 biogenically active amines in tea leaves, infusions, and tea drinks. Optimization of sample extraction and clean-up produced good analytical performances in terms of recovery, linearity, precision, and sensitivity. In particular TCA 5% was demonstrated to be the best analytical choice for optimum extraction of BAs from tea, although considering that water tea infusions and beverages are actually ingested by humans, only water-extractable BAs are of dietary importance.

As can be noted from reported data, it can be concluded that tea and tea infusions in particular do not represent a possible risk for consumer health considering the existing and/or suggested limits for BAs in foods. Anyway it should be considered that there are many other dietary sources of BAs. In this sense, improving the knowledge of BAs concentrations in relation to agricultural practice (organic farming in particular) and processing will provide a more accurate insight in parameters affecting BAs formation in the final product, leading to a more precise estimate of BAs intake from foods.

## Figures and Tables

**Table 1:** Main characteristics of tea samples.

Sample	Kind of tea	Origin	Cultivation	Caffeine content (w/w%)
1	Black	Kenia	Conventional	1.5
2	Black	China	Conventional	1.7
3	Black	China	Conventional	1.5
4	Black	Tanzania	Conventional	1.6
5	Black	Tanzania	Conventional	1.5
6	Black	China	Conventional	1.8
7	Black	India	Conventional	1.6
8	Black	Sri Lanka	Organic	1.9
9	Black	Kenia	Conventional	≤0.1%
10	Black	India	Conventional	≤0.1%
11	Black	India	Conventional	≤0.1%
12	Black	China	Conventional	≤0.1%
13	Black	China	Conventional	≤0.1%
14	Black	Kenia	Organic	1.7
15	Green	China	Conventional	2.0
16	Green	Tanzania	Conventional	≤0.1%
17	Green	India	Conventional	1.8
18	Green	Sri Lanka	Conventional	≤0.1%
19	Green	Sri Lanka	Conventional	2.0
20	Green	India	Organic	1.6
21	Green	Kenia	Organic	1.7

**Table 2:** LC-UV method performance (*n* = 6).

BA	Extraction solvents																				
	HCl 0.1 M							HClO_4_ 0.1 M							TCA 5% (w/w)						
	*R* ^2^	LOD^a^ (*μ*g L^−1^)	LOQ^a^ (*μ*g L^−1^)	LOD^b^ (*μ*g g^−1^)	LOQ^b^ (*μ*g g^−1^)	LOD^c^ (*μ*g L^−1^)	LOQ^c^ (*μ*g L^−1^)	*R* ^2^	LOD^a^ (*μ*g L^−1^)	LOQ^a^ (*μ*g L^−1^)	LOD^b^ (mg Kg^−1^)	LOQ^b^ (mg Kg^−1^)	LOD^c^ (*μ*g L^−1^)	LOQ^c^ (*μ*g L^−1^)	*R* ^2^	LOD^a^ (*μ*g L^−1^)	LOQ^a^ (*μ*g L^−1^)	LOD^b^ (mg Kg^−1^)	LOQ^b^ (mg Kg^−1^)	LOD^c^ (*μ*g L^−1^)	LOQ^c^ (*μ*g L^−1^)
PHE	0.99	0.29	0.91	0.41	1.08	0.31	0.92	0.99	0.30	0.90	0.40	1.11	0.32	0.95	0.99	0.27	0.90	0.40	1.07	0.30	0.90
PUT	0.99	0.11	0.30	0.25	0.80	0.18	0.52	0.99	0.10	0.32	0.27	0.82	0.19	0.53	0.99	0.10	0.29	0.23	0.70	0.18	0.50
CAD	0.99	0.19	0.60	0.35	1.09	0.32	0.98	0.99	0.20	0.58	0.36	1.10	0.33	0.99	0.99	0.17	0.59	0.33	1.08	0.30	0.97
HIS	0.99	0.30	0.92	0.40	1.03	0.32	0.95	0.99	0.28	0.90	0.41	1.05	0.33	0.97	0.99	0.29	0.90	0.35	1.00	0.31	0.94
TYR	0.99	0.41	1.28	0.45^*∗*^	1.31^*∗*^	0.43^*∗*^	1.30^*∗*^	0.99	0.42	1.26	0.46^*∗*^	1.35^*∗*^	0.45^*∗*^	1.33^*∗*^	0.99	0.40	1.27	0.40^*∗*^	1.30^*∗*^	0.42^*∗*^	1.28^*∗*^
SPD	0.99	0.27	0.72	0.32	0.83	0.29	0.76	0.99	0.29	0.70	0.31	0.82	0.30	0.78	0.99	0.26	0.70	0.30	0.80	0.27	0.75
SER	0.99	0.24	0.78	0.30	0.90	0.26	0.84	0.99	0.26	0.75	0.32	0.92	0.27	0.85	0.99	0.23	0.77	0.29	0.88	0.25	0.83
SPM	0.99	0.25	0.60	0.27	0.75	0.30	0.81	0.99	0.24	0.61	0.29	0.76	0.32	0.86	0.99	0.24	0.59	0.24	0.70	0.29	0.80

^*∗*^TYR was added (100 *μ*L 0.05 mg mL^−1^); ^a^BA standard solution; ^b^tea leaves; ^c^tea infusion. PHE, *β*-phenylethylamine; PUT, putrescine; CAD, cadaverine; HIS, histamine; TYR, tyramine; SPD, spermidine; SER, serotonin; SPM, spermine. TCA, trichloroacetic acid, LOD, limit of detection; LOQ, limit of quantitation.

**Table 3:** Values of recovery, coefficient of variation, and intra- and interday repeatability obtained with LC-UV on tea leaves.

BA	HCl 0.1 M				HClO_4_ 0.1 M				TCA 5% (w/w)			
	Recovery (%)	CV	RSD intraday	RSD interday	Recovery (%)	CV	RSD intraday	RSD interday	Recovery (%)	CV	RSD intraday	RSD interday
PHE	90.4	6.8	0.3	0.4	91.4	6.9	0.3	0.4	98.4	4.7	0.2	0.3
PUT	92.3	4.1	0.3	0.4	88.3	4.1	0.3	0.4	98.3	4.1	0.1	0.2
CAD	94.0	4.4	0.3	0.4	94.2	4.5	0.3	0.4	96.5	1.4	0.1	0.2
HIS	92.5	4.9	0.3	0.4	87.5	4.3	0.3	0.4	97.5	2.4	0.2	0.3
TYR	91.0	—	0.2	0.3	89.0	—	0.2	0.3	98.0	—	0.1	0.2
SPD	89.5	4.6	0.4	0.4	88.5	4.9	0.3	0.4	98.5	2.4	0.1	0.2
SER	88.0	6.0	0.4	0.4	87.8	6.1	0.4	0.4	98.9	1.9	0.1	0.2
SPM	94.0	6.0	0.3	0.4	93.0	4.8	0.3	0.4	98.0	2.4	0.2	0.3

2.0 g of sample 5 was spiked with 1.0 mL of a BAs standard solution at concentration of 2.0 mg L^−1^.

PHE, *β*-phenylethylamine; PUT, putrescine; CAD, cadaverine; HIS, histamine; TYR, tyramine; SPD, spermidine; SER, serotonin; SPM, spermine; TCA: trichloroacetic acid.

**Table 4:** Values of recovery, coefficient of variation, and intra- and interday repeatability obtained with LC-UV on tea infusions.

AB	HCl 0.1 M				HClO_4_ 0.1 M				TCA 5% (w/w)			
	Recovery (%)	CV	RSD intraday	RSD interday	Recovery (%)	CV	RSD intraday	RSD interday	Recovery (%)	CV	RSD intraday	RSD interday
PHE	90.4	—	0.3	0.4	91.4	—	0.3	0.4	97.4	—	0.1	0.3
PUT	92.3	2.7	0.3	0.4	88.3	2.6	0.3	0.4	98.8	2.6	0.1	0.2
CAD	93.0	6.2	0.3	0.4	94.5	6.4	0.3	0.4	97.5	2.2	0.2	0.3
HIS	90.5	4.3	0.3	0.3	87.5	4.5	0.3	0.3	97.6	2.2	0.1	0.2
TYR	90.0	—	0.2	0.3	88.0	—	0.2	0.3	99.0	—	0.1	0.2
SPD	88.5	5.8	0.4	0.4	88.5	6.0	0.4	0.4	97.5	2.8	0.1	0.2
SER	87.0	10.0	0.4	0.5	86.8	9.5	0.4	0.5	99.9	3.5	0.1	0.2
SPM	94.1	7.8	0.3	0.4	95.0	6.6	0.3	0.4	98.0	4.8	0.1	0.2

40 mL of sample 5 was spiked with 5.0 mL of a BAs standard solution at concentration of 2.0 mg L^−1^.

PHE, *β*-phenylethylamine; PUT, putrescine; CAD, cadaverine; HIS, histamine; TYR, tyramine; SPD, spermidine; SER, serotonin; SPM, spermine; TCA: trichloroacetic acid.

**Table 5:** Biogenic amines content in tea leaves. Data represent mean ± RSD (*n* = 3); *p* < 0.05.

Sample	Biogenic amines (*μ*g g^−1^)								
	PHE	PUT	CAD	HIS	TYR	SPD	SER	SPM	TOT
1^a^	ND	1.62 ± 0.07	ND	1.91 ± 0.05	ND	1.83 ± 0.08	1.09 ± 0.01	ND	6.45 ± 0.07
2^a^	ND	1.27 ± 0.06	ND	2.44 ± 0.09	ND	1.81 ± 0.04	1.03 ± 0.01	ND	6.55 ± 0.07
3^a^	ND	1.65 ± 0.07	ND	2.20 ± 0.06	ND	1.15 ± 0.06	1.07 ± 0.01	ND	6.07 ± 0.06
4^a^	0.98 ± 0.05	1.06 ± 0.01	1.19 ± 0.01	1.69 ± 0.03	ND	1.51 ± 0.03	1.02 ± 0.01	ND	7.45 ± 0.03
5^a^	1.28 ± 0.06	1.97 ± 0.08	1.40 ± 0.02	1.69 ± 0.04	ND	1.70 ± 0.04	1.56 ± 0.03	1.64 ± 0.04	11.24 ± 0.09
6^a^	1.21 ± 0.06	1.10 ± 0.01	1.30 ± 0.02	ND	ND	1.48 ± 0.03	1.04 ± 0.01	ND	6.13 ± 0.03
7^a^	ND	1.84 ± 0.04	1.41 ± 0.02	1.71 ± 0.04	ND	1.53 ± 0.03	1.08 ± 0.01	1.59 ± 0.03	9.16 ± 0.05
8^a^	ND	1.30 ± 0.01	ND	ND	ND	1.39 ± 0.03	1.52 ± 0.01	1.25 ± 0.02	5.46 ± 0.02
9^a^	2.52 ± 0.07	1.30 ± 0.06	ND	ND	ND	1.09 ± 0.07	ND	ND	4.91 ± 0.08
10^a^	ND	1.56 ± 0.07	ND	1.71 ± 0.07	ND	1.01 ± 0.04	ND	0.92 ± 0.05	5.20 ± 0.08
11^a^	ND	1.21 ± 0.01	ND	1.69 ± 0.04	ND	1.44 ± 0.02	ND	ND	4.34 ± 0.02
12^a^	ND	2.25 ± 0.09	ND	ND	ND	1.66 ± 0.04	ND	1.47 ± 0.03	5.38 ± 0.04
13^a^	ND	1.41 ± 0.02	ND	ND	ND	1.95 ± 0.05	ND	0.93 ± 0.05	4.29 ± 0.03
14^a^	ND	1.05 ± 0.01	ND	1.69 ± 0.05	ND	1.60 ± 0.05	0.81 ± 0.01	0.74 ± 0.05	5.89 ± 0.03
15^b^	ND	1.29 ± 0.02	ND	ND	ND	1.74 ± 0.08	0.99 ± 0.1	ND	4.02 ± 0.09
16^b^	ND	1.50 ± 0.07	ND	ND	ND	1.28 ± 0.06	1.06 ± 0.05	ND	3.84 ± 0.05
17^b^	ND	1.26 ± 0.03	ND	ND	ND	1.25 ± 0.08	1.24 ± 0.04	ND	3.75 ± 0.03
18^b^	ND	1.08 ± 0.01	ND	ND	ND	1.15 ± 0.02	ND	ND	2.23 ± 0.01
19^b^	ND	1.44 ± 0.01	ND	ND	ND	1.90 ± 0.02	0.83 ± 0.01	ND	4.17 ± 0.01
20^b^	ND	1.06 ± 0.01	ND	ND	ND	1.15 ± 0.03	1.05 ± 0.01	ND	3.26 ± 0.01
21^b^	ND	1.09 ± 0.01	ND	ND	ND	1.09 ± 0.03	1.38 ± 0.02	ND	3.56 ± 0.01

^a^Black tea; ^b^green tea. PHE, *β*-phenylethylamine; PUT, putrescine; CAD, cadaverine; HIS, histamine; TYR, tyramine; SPD, spermidine; SER, serotonin; SPM, spermine.

**Table 6:** Biogenic amines content in tea infusions. Data represent mean ± RSD (*n* = 3); *p* < 0.05.

Sample	Biogenic amines$\begin{array}{c} \left(\mu g\;L^{-1}\right)\\ \left(\mu g\;g^{-1}\right) \end{array}$								
	PHE	PUT	CAD	HIS	TYR	SPD	SER	SPM	TOT
1^a^	ND	14.9 ± 0.5	ND	15.3 ± 0.4	ND	10.6 ± 0.3	9.6 ± 0.1	ND	50.4 ± 0.5
		0.75 ± 0.02		0.77 ± 0.02		0.53 ± 0.03	0.48 ± 0.02		2.53 ± 0.02

2^a^	ND	12.7 ± 0.3	ND	20.0 ± 0.4	ND	10.8 ± 0.2	9.1 ± 0.2	ND	52.6 ± 0.4
		0.64 ± 0.02		1.0 ± 0.05		0.54 ± 0.02	0.46 ± 0.02		2.64 ± 0.03

3^a^	ND	16.0 ± 0.6	ND	17.3 ± 0.4	ND	7.0 ± 0.4	9.4 ± 0.2	ND	49.7 ± 0.7
		0.80 ± 0.03		0.87 ± 0.04		0.35 ± 0.02	0.47 ± 0.02		2.49 ± 0.02

4^a^	ND	10.2 ± 0.5	11.8 ± 0.3	13.0 ± 0.4	ND	9.4 ± 0.3	9.3 ± 0.2	ND	53.7 ± 0.6
		0.51 ± 0.02	0.59 ± 0.02	0.65 ± 0.03		0.47 ± 0.02	0.47 ± 0.02		2.69 ± 0.02

5^a^	ND	19.3 ± 0.5	13.5 ± 0.3	12.8 ± 0.3	ND	10.7 ± 0.4	14.1 ± 0.5	10.3 ± 0.5	80.7 ± 0.9
		0.96 ± 0.04	0.68 ± 0.03	0.64 ± 0.03		0.54 ± 0.02	0.71 ± 0.04	0.52 ± 0.03	4.05 ± 0.04

6^a^	ND	11.1 ± 0.2	12.4 ± 0.3	ND	ND	8.8 ± 0.2	9.1 ± 0.5	ND	41.4 ± 0.2
		0.56 ± 0.02	0.62 ± 0.03			0.44 ± 0.03	0.46 ± 0.03		2.08 ± 0.02

7^a^	ND	18.8 ± 0.2	14.0 ± 0.2	14.1 ± 0.3	ND	9.2 ± 0.2	9.3 ± 0.1	9.9 ± 0.3	75.3 ± 0.3
		0.94 ± 0.03	0.70 ± 0.04	0.71 ± 0.02		0.46 ± 0.03	0.47 ± 0.02	0.50 ± 0.02	3.78 ± 0.03

8^a^	ND	16.0 ± 0.2	ND	ND	ND	8.2 ± 0.3	12.9 ± 0.2	8.8 ± 0.3	45.9 ± 0.2
		0.70 ± 0.03				0.46 ± 0.03	0.66 ± 0.04	0.49 ± 0.02	2.31 ± 0.03

9^a^	2.0 ± 0.3	12.9 ± 0.3	ND	ND	ND	7.0 ± 0.2	ND	ND	21.9 ± 0.3
	0.10 ± 0.01	0.64 ± 0.02				0.35 ± 0.02			1.09 ± 0.01

10^a^	ND	15.0 ± 0.2	ND	13.0 ± 0.3	ND	6.5 ± 0.2	ND	5.5 ± 0.3	40.0 ± 0.2
		0.75 ± 0.02		0.65 ± 0.02		0.33 ± 0.02		0.28 ± 0.01	2.01 ± 0.02

11^a^	ND	12.3 ± 0.2	ND	14.0 ± 0.3	ND	8.3 ± 0.2	ND	ND	34.6 ± 0.2
		0.62 ± 0.02		0.70 ± 0.03		0.42 ± 0.03			1.74 ± 0.02

12^a^	ND	21.0 ± 0.5	ND	ND	ND	10.0 ± 0.2	ND	8.9 ± 0.3	39.9 ± 0.2
		1.1 ± 0.05				0.50 ± 0.03		0.45 ± 0.02	2.05 ± 0.03

13^a^	ND	13.8 ± 0.2	ND	ND	ND	11.1 ± 0.3	ND	5.2 ± 0.3	30.1 ± 0.3
		0.69 ± 0.02				0.56 ± 0.04		0.26 ± 0.01	1.51 ± 0.02

14^a^	ND	8.4 ± 0.2	ND	11.1 ± 0.3	ND	8.8 ± 0.3	7.5 ± 0.1	5.0 ± 0.4	40.8 ± 0.6
		0.47 ± 0.02		0.60 ± 0.03		0.44 ± 0.04	0.48 ± 0.02	0.30 ± 0.02	2.29 ± 0.03

15^b^	ND	12.0 ± 0.9	ND	ND	ND	10.4 ± 0.5	9.5 ± 0.7	ND	31.9 ± 0.8
		0.60 ± 0.03				0.52 ± 0.03	0.48 ± 0.03		1.60 ± 0.03

16^b^	ND	15.4 ± 1.2	ND	ND	ND	7.3 ± 0.3	9.6 ± 0.3	ND	32.3 ± 0.8
		0.77 ± 0.03				0.37 ± 0.02	0.48 ± 0.03		1.62 ± 0.02

17^b^	ND	12.6 ± 0.2	ND	ND	ND	7.3 ± 0.4	10.5 ± 0.2	ND	30.4 ± 0.2
		0.63 ± 0.02				0.37 ± 0.03	0.53 ± 0.03		1.53 ± 0.02

18^b^	ND	10.5 ± 0.1	ND	ND	ND	6.3 ± 0.3	ND	ND	16.8 ± 0.3
		0.53 ± 0.02				0.32 ± 0.02			0.85 ± 0.02

19^b^	ND	14.6 ± 0.1	ND	ND	ND	10.3 ± 0.4	8.0 ± 0.3	ND	32.9 ± 0.2
		0.73 ± 0.03				0.52 ± 0.02	0.40 ± 0.02		1.65 ± 0.02

20^b^	ND	10.3 ± 0.2	ND	ND	ND	6.7 ± 0.5	9.7 ± 0.5	ND	26.7 ± 0.6
		0.52 ± 0.02				0.34 ± 0.02	0.49 ± 0.02		1.35 ± 0.02

21^b^	ND	10.7 ± 0.2	ND	ND	ND	6.3 ± 0.3	11.5 ± 0.3	ND	28.5 ± 0.2
		0.54 ± 0.02				0.32 ± 0.02	0.58 ± 0.03		1.44 ± 0.02

22^c^	ND	6.9 ± 0.1	ND	ND	ND	5.5 ± 0.2	ND	ND	12.4 ± 0.1

23^c^	ND	ND	ND	ND	ND	4.3 ± 0.2	ND	ND	4.3 ± 0.1

24^c^	ND	ND	ND	ND	ND	6.7 ± 0.2	ND	ND	6.7 ± 0.1

^a^Black tea; ^b^green tea; ^c^tea drink. PHE, *β*-phenylethylamine; PUT, putrescine; CAD, cadaverine; HIS, histamine; TYR, tyramine; SPD, spermidine; SER, serotonin; SPM, spermine; ND, not detectable.

## References

[1] Food and Agriculture Organizaton of the United Nations (FAO) http://faostat.fao.org/.

[2] Zhao J., Chen Q., Huang X., Fang C. H. (2006). Qualitative identification of tea categories by near infrared spectroscopy and support vector machine. *Journal of Pharmaceutical and Biomedical Analysis*.

[3] Hayat K., Iqbal H., Malik U., Bilal U., Mushtaq S. (2015). Tea and its consumption: benefits and risks. *Critical Reviews in Food Science and Nutrition*.

[4] Vuong Q. V. (2014). Epidemiological evidence linking tea consumption to human health: a review. *Critical Reviews in Food Science and Nutrition*.

[5] Teti D., Visalli M., McNair H. (2002). Analysis of polyamines as markers of (patho)physiological conditions. *Journal of Chromatography B*.

[6] Silla Santos M. H. (1996). Biogenic amines: their importance in foods. *International Journal of Food Microbiology*.

[7] European Food Safety Authority (EFSA) (2011). Scientific opinion on risk based control of biogenic amine formation in fermented foods. *EFSA Journal*.

[8] Granvogl M., Bugan S., Schieberle P. (2006). Formation of amines and aldehydes from parent amino acids during thermal processing of cocoa and model systems: new insights into pathways of the strecker reaction. *Journal of Agricultural and Food Chemistry*.

[9] Oracz J., Nebesny E. (2014). Influence of roasting conditions on the biogenic amine content in cocoa beans of different Theobroma cacao cultivars. *Food Research International*.

[10] Zamora R., Delgado R. M., Hidalgo F. J. (2012). Formation of *β*-phenylethylamine as a consequence of lipid oxidation. *Food Research International*.

[11] Bardócz S. (1995). Polyamines in food and their consequences for food quality and human health. *Trends in Food Science and Technology*.

[12] Önal A., Tekkeli S. E. K., Önal C. (2013). A review of the liquid chromatographic methods for the determination of biogenic amines in foods. *Food Chemistry*.

[13] Önal A. (2007). A review: current analytical methods for the determination of biogenic amines in foods. *Food Chemistry*.

[14] Restuccia D., Spizzirri U. G., Puoci F. (2011). A new method for the determination of biogenic amines in cheese by LC with evaporative light scattering detector. *Talanta*.

[15] European Commission

[16] Custódio F. B., Tavares É., Glória M. B. A. (2007). Extraction of bioactive amines from grated Parmesan cheese using acid, alkaline and organic solvents. *Journal of Food Composition and Analysis*.

[17] Korös Á., Varga Z., Molnár-Perl I. (2008). Simultaneous analysis of amino acids and amines as their *o*-phthalaldehyde-ethanethiol-9-fluorenylmethyl chloroformate derivatives in cheese by high-performance liquid chromatography. *Journal of Chromatography A*.

[18] Okamoto A., Sugi E., Koizumi Y., Yanagida F., Udaka S. (1997). Polyamine content of ordinary foodstuffs and various fermented foods. *Bioscience, Biotechnology, and Biochemistry*.

[19] Palavan-Ünsal N., Arisan E. D., Terzioglu S. (2007). Polyamines in tea processing. *International Journal of Food Sciences and Nutrition*.

[20] Sun J.-S., Guo H.-X., Semin D., Cheetham J. (2011). Direct separation and detection of biogenic amines by ion-pair liquid chromatography with chemiluminescent nitrogen detector. *Journal of Chromatography A*.

[21] Brückner H., Flassig S., Kirschbaum J. (2012). Determination of biogenic amines in infusions of tea (Camellia sinensis) by HPLC after derivatization with 9-fluorenylmethoxycarbonyl chloride (FMOC-Cl). *Amino Acids*.

[22] Martuscelli M., Gardini F., Torriani S. (2005). Production of biogenic amines during the ripening of Pecorino Abruzzese cheese. *International Dairy Journal*.

[23] Zhang M. Y., Fan H., Fu Z. (2014). Determination of eight different biogenic amines in pu'er tea by HPLC. *Focusing on Modern Food Industry*.

[24] Lapa-Guimarães J., Pickova J. (2004). New solvent systems for thin-layer chromatographic determination of nine biogenic amines in fish and squid. *Journal of Chromatography A*.

[25] Novella-Rodríguez S., Teresa Veciana-Nogués M., Carmen Vidal-Carou M. (2000). Biogenic amines and polyamines in milks and cheeses by ion-pair high performance liquid chromatography. *Journal of Agricultural and Food Chemistry*.

[26] Bailey S. R., Marr C. M., Elliott J. (2003). Identification and quantification of amines in the equine caecum. *Research in Veterinary Science*.

[27] Mazzucco E., Gosetti F., Bobba M., Marengo E., Robotti E., Gennaro M. C. (2010). High-performance liquid chromatography-ultraviolet detection method for the simultaneous determination of typical biogenic amines and precursor amino acids: applications in food chemistry. *Journal of Agricultural and Food Chemistry*.

[28] Codex Alimentarius (1993). *Residuos de Medicamentos Veterinarios en los Alimentos*.

[29] Larqué E., Sabater-Molina M., Zamora S. (2007). Biological significance of dietary polyamines. *Nutrition*.

[30] Kalač P., Krausová P. (2005). A review of dietary polyamines: formation, implications for growth and health and occurrence in foods. *Food Chemistry*.

[31] Kalač P. (2014). Health effects and occurrence of dietary polyamines: a review for the period 2005-mid 2013. *Food Chemistry*.

[32] Restuccia D., Spizzirri U. G., Parisi O. I., Cirillo G., Picci N. (2015). Brewing effect on levels of biogenic amines in different coffee samples as determined by LC-UV. *Food Chemistry*.

[33] Loizzo M. R., Menichini F., Picci N., Puoci F., Spizzirri U. G., Restuccia D. (2013). Technological aspects and analytical determination of biogenic amines in cheese. *Trends in Food Science and Technology*.

[34] Spizzirri U. G., Parisi O. I., Picci N., Restuccia D. (2016). Application of LC with evaporative light scattering detector for biogenic amines determination in fair trade cocoa-based products. *Food Analytical Methods*.

[35] Leite da Silveira T. M., Tavares É., Glória M. B. A. (2007). Profile and levels of bioactive amines in instant coffee. *Journal of Food Composition and Analysis*.

[36] Restuccia D., Spizzirri U. G., Puoci F., Picci N. (2015). Determination of biogenic amine profiles in conventional and organic cocoa-based products. *Food Additives and Contaminants—Part A: Chemistry, Analysis, Control, Exposure and Risk Assessment*.

[37] Han W.-Y., Xu J.-M., Wei K., Shi R.-Z., Ma L.-F. (2013). Soil carbon sequestration, plant nutrients and biological activities affected by organic farming system in tea (*Camellia sinensis* (L.) O. Kuntze) fields. *Soil Science and Plant Nutrition*.

[38] Nishimura K., Shiina R., Kashiwagi K., Igarashi K. (2006). Decrease in polyamines with aging and their ingestion from food and drink. *Journal of Biochemistry*.

[39] Ingles D. L., Back J. F., Gallimore D., Tindale R., Shaw K. J. (1985). Estimation of biogenic amines in foods. *Journal of the Science of Food and Agriculture*.

[40] Casal S., Mendes E., Alves M. R. (2004). Free and conjugated biogenic amines in green and roasted coffee beans. *Journal of Agricultural and Food Chemistry*.

[41] Kakkar R. K., Nagar P. K. (1997). Distribution and changes in endogenous polyamines during winter dormancy in tea [*Camellia sinensis* L. (O) kuntze]. *Journal of Plant Physiology*.

